# Bayesian Analysis of Length of Stay Determinants in ERAS-Guided Hip Arthroplasty

**DOI:** 10.3390/healthcare13070777

**Published:** 2025-03-31

**Authors:** Nan Yao, Xiaoyan Wang, Meng Yang, Xinglei Wang, Xinman Dou

**Affiliations:** 1 School of Nursing, Lanzhou University, Lanzhou 730000, China; yaon2023@lzu.edu.cn (N.Y.);; 2 Department of Nursing, The Second Hospital of Lanzhou University, Lanzhou 730030, China

**Keywords:** enhanced recovery after surgery, total hip arthroplasty, hospitalization

## Abstract

**Background and Objectives**: Total hip arthroplasty in China expanded rapidly post-2019. The length of hospital stay in these procedures reflects healthcare quality standards. This study analyzed the correlation between preoperative clinical factors and the length of hospital stay in total hip arthroplasty patients managed via an enhanced recovery after surgery protocol. **Methods**: Preoperative clinical variables were collected from total hip arthroplasty patients in an accelerated rehabilitation program. One-way ANOVA and other statistical methods analyzed correlations between these data and hospitalization time. **Results**: A total of 408 patients were included, with a mean length of stay of 12.01 ± 4.281 days. Right lower extremity strength (*t* = 2.794, *p* = 0.005), activities of daily living score (*t* = −3.481, *p* = 0.001), C-reactive protein (*t* = −2.514, *p* = 0.016), thrombin time (*t* = −2.393, *p* = 0.019), and prothrombin activity (*t* = 2.582, *p* = 0.013) can directly affect the length of stay in patients with total hip arthroplasty. Also, age (*F* = 1.958, *p* = 0.006) and erythrocyte sedimentation rate (*t* = −2.519, *p* = 0.015) were found to affect the length of hospital stay indirectly. **Conclusions**: This study demonstrated that right lower extremity strength, activities of daily living score, C-reactive protein, thrombin time, and prothrombin activity significantly influence the length of hospital stay in enhanced recovery after surgery-managed total hip arthroplasty patients. Therefore, early interventions should be made to address the above factors.

## 1. Introduction

The escalating osteoarthritis burden in Asia is evidenced by arthroplasty prevalence trends, with China performing 950,000 total hip arthroplasties (THAs)/total knee arthroplasties (TKAs) in 2019 [1]—a 330% increase from 2011—contrasting Japan’s 146,000 annual cases [2,3]. Accelerated population aging (≥65 years) coupled with metabolic syndrome epidemics (in South and Southeast Asia, the prevalence of obesity is predicted to double between 2010 and 2030 [4]) is driving exponential growth in THA demand through the mid-century, particularly in Asia [5]. As the gold-standard intervention for terminal hip degeneration, THA achieves the primary therapeutic objectives of pain mitigation and functional recovery [6]. Existing evidence confirms the secondary rehabilitation benefits of THA. These benefits show measurable improvements in quality of life [7,8]. They also demonstrate systematic progress in daily activity capabilities [9,10].

The enhanced recovery after surgery (ERAS) practice was first developed in the 1990s. It combines evidence-based, multidisciplinary methods to reduce surgical stress and speed recovery [11]. The ERAS Society has promoted the systematic use of perioperative care programs (“fast-track” or “accelerated recovery pathways”) for nearly 20 years. Clinical practice shows ERAS protocols significantly shorten the length of stay (LOS) [12]. Some patients even achieve same-day discharge (SDD) [13]. These approaches also reduce patients’ financial burdens [14].

Linear regression analysis (LRA) was historically the dominant method for studying LOS predictors [15]. It analyzes variable correlations, but cannot distinguish direct/indirect influences, limiting clinical utility. Bayesian networks (BNs) address this limitation. BNs have two components: directed acyclic graphs (DAGs) and conditional probability tables (CPTs). They model complex variable interactions and enable probabilistic reasoning under uncertainty [16,17,18]. BNs leverage preoperative data to infer LOS probability and flexibly, showing the influence of influencing factors on LOS. BN learning refers to obtaining a complete BN from the available information, and it is constructed by parameter learning and structure learning [19]. Structure learning, central to a BN, combines score-based and constraint-based methods, but faces challenges in network optimization. The hybrid max–min hill-climbing (MMHC) algorithm integrates these approaches to enhance accuracy [20].

Established predictors of post-arthroplasty LOS include patient-specific factors (age, BMI, and comorbidities) [21,22,23,24], pre-operative functional assessments (ASA scores and PROMs) [25], and institutional protocols (surgical technique and ERAS compliance) [26,27,28]. Preoperative biochemical markers show sporadic associations with outcomes. Examples include isolated indicators like C-reactive protein and albumin [29,30]. However, these markers remain systematically absent from LOS prediction models. This study directly investigated preoperative biochemical profiles to address this evidence gap.

This two-phase study first employed LRA to identify LOS determinants in THA patients. Subsequently, a BN framework was developed using the MMHC algorithm. This framework integrated constraint-based pruning and score-based optimization. It established directional relationships between variables to guide LOS management [31].

## 2. Materials and Methods

### 2.1. Design

This retrospective study analyzed THA patients managed via ERAS protocols at our institution (2018–2024). Patient records were systematically reviewed to identify LOS determinants.

### 2.2. Study Setting and Sampling

Data were collected retrospectively from the hospital information system (HIS) of the Second Hospital of Lanzhou University. Data were collected from 1 January 2018 to 1 January 2024. This study included 408 patients, including 204 female patients.

### 2.3. Inclusion and Exclusion Criteria

Participants in this study were recruited from patients who underwent THA with hip osteoarthritis. Inclusion criteria for this study were as follows: (1) adults over 18 years of age; (2) admitted to the hospital for osteoarthritis; (3) no communication disorders; (4) willingness to participate in this study; and (5) primary THA. Exclusion criteria included (1) previous history of psychiatric or cognitive dysfunction and (2) surgery other than THA during current hospitalization.

### 2.4. Data Source

Patient data were retrieved from institutional electronic health records via the HIS.

### 2.5. Data Collection and Data Analysis

Data in this study included hospitalization registration number, sex, age, time of surgery, LOS, left upper extremity strength, left lower extremity strength, right upper extremity strength, right lower extremity strength, activities of daily living score (ADL score), fall risk score, Branden score, C-reactive protein, urine specific gravity, prothrombin activity, international normalized ratio, thrombin time, D-dimer, white blood cell count, red blood cell count, hemoglobin count, platelet count, glucose, creatinine, carbamide, uric acid, blood potassium, total protein, erythrocyte sedimentation rate (ESR), and interleukin-6. Preoperative laboratory parameters were collected via standard fasting venous blood and urine samples. This collection occurred within 24 h before surgery. Nurses measured muscle strength and ADL scores. These measurements occurred on the morning of the surgery, before the operation.

A priori power analysis (PASS 2021) suggested N = 241 was needed to detect *f*^2^ = 0.15 (Δ*R*^2^ = 0.15) with k = 7 predictors (*α* = 0.05, 80% power). Post-hoc analysis of collected data (N = 408) showed 97% power to detect Δ*R*^2^ = 0.055 from the predictors after accounting for covariates ($R_{\mathrm{cov}}^{2}$ = 0.05), equivalent to *f*^2^ = 0.058. Statistical analyses employed SPSS 27.0 (univariate/multivariate) with categorical conversions of continuous variables (*p* < 0.05 significance threshold). BN modeling utilized R’s “bnlearn” package (Version 4.3.3), implementing the MMHC algorithm for DAG structure learning and maximum likelihood estimation for parameter optimization. Network visualization and CPT generation were executed in Netica (Version 6.9.0.0). 

### 2.6. Ethical Approval

The Ethics Committee of the Second Hospital of Lanzhou University granted ethical approval for this retrospective study (Approval No. 2024A-682).

## 3. Results

### 3.1. Characteristics of Patients

A total of 408 patients who met the inclusion criteria and did not meet the exclusion criteria for primary THA were enrolled in this study, including 204 men and 204 women, each representing 50% of the total. D-dimer levels demonstrated the highest missing data rate (40.9%, n = 167/408). Demographic data (age, sex) maintained complete integrity. Among the ages < 41 years old, 41–60 years old, and > 60 years old, the proportions were 16.0%, 60.0%, and 24.0%, respectively. The proportions of ADL scores of 100, 61–99, 41–60, and < 41 were 22.06%, 66.42%, 6.13%, and 3.43%, respectively. The average length of hospitalization was 12.01 ± 4.281 days. Additional details can be found in Table S1 of the Supplementary Document.

### 3.2. Univariate Analysis of LOS for Primary THA Recipients

Age, right lower extremity muscle strength, ADL score, C-reactive protein, thrombin time, prothrombin activity, and ESR were all statistically significant (*p* < 0.05), as shown in Table 1. Additional details can be found in Table S1 of the Supplementary Document.

### 3.3. Multifactorial Analysis of LOS for Primary THA Recipients

The LOS for primary THA was the dependent variable. All general information served as independent variables in the regression analysis. Right lower extremity strength, ADL score, and ESR significantly influenced LOS in primary THA (Table 2). Supplementary Table S1 contains additional detailed data.

### 3.4. Building a BN

**Figure 1:**
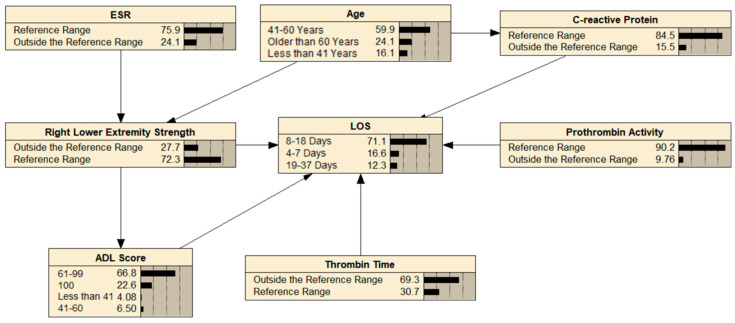
MMHC algorithm to construct a BN of factors influencing LOS for THA patients.

All statistically significant variables were converted into categorical variables. These variables were used as network nodes to build a BN model of factors affecting LOS in primary THA patients (Figure 1) and to calculate node probabilities.

### 3.5. BN Reasoning

**Figure 2:**
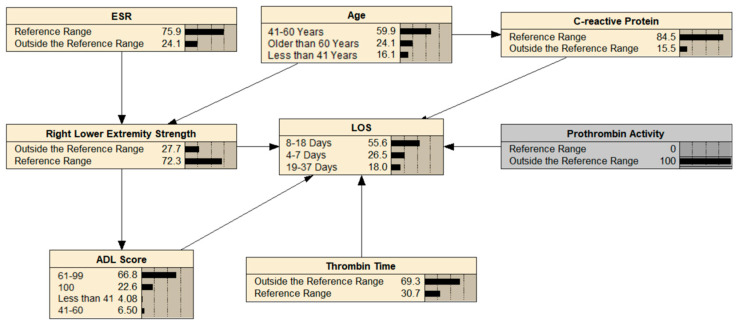
BN’s reasoning for LOS in THA patients.

The BN could infer unknown nodes from known nodes, allowing LOS to be predicted. As shown in Figure 2, the probability that a patient’s LOS was > 18 days when prothrombin activity was outside the reference range increased from 12.3% to 18.0%.

## 4. Discussion

We found that right lower extremity strength, ADL score, C-reactive protein, thrombin time, and prothrombin activity can directly influence LOS in patients with THA. Also, age and ESR were found to influence LOS indirectly. Our right lower extremity strength results agree with those of LeBrun et al., who noted that higher lower extremity mobility before surgery predicted a shorter LOS [32]. Also, the results of ADL scores agree with those of Vasta and Hewlett-Smith et al., who noted that the higher the ADL, the shorter the LOS [33,34]. In terms of C-reactive protein, our results are in agreement with those of Missmann et al. Higher C-reactive protein values are associated with a longer LOS [23]. In addition, we found that thrombin time slightly affected LOS, whereas prothrombin activity significantly prolonged LOS. Regarding indirect influences, both our study and that of Stone et al. state that age prolongs LOS [35]. Meanwhile, our research and Li et al. demonstrated that ESR influences LOS [36]; however, we further clarified the specific pathway through which ESR affects LOS, specifically via its indirect effect on the muscle strength of the right lower extremity.

In the present study, right lower extremity strength was an independent predictor of LOS, and the mechanism may involve multifactorial effects. An elevated ESR may limit hip mobility and reduce lower limb muscle strength by exacerbating synovial inflammation and pain [37,38]. Patients are already malnourished preoperatively, which directly reduces the rate of muscle protein synthesis [39], and hypercortisolism occurs during the surgical procedure, with a reduction in protein synthesis and an increase in protein catabolism, which ultimately results in a significant loss of skeletal muscle [40], resulting in lower limb muscle strength loss, which in turn prolongs the recovery time of postoperative mobility [41]. In addition, right lower extremity strength deficits directly affect ADL scores (e.g., walking, toileting, and other activities that depend on lower extremity muscle groups) [41], and thus low ADL scores comprehensively reflect the association between muscle strength deficits and LOS prolongation. Meanwhile, the effect of elevated C-reactive protein on LOS is dual: on the one hand, preoperative C-reactive protein > 5 mg/L suggests an elevated risk of infection [42,43]; on the other hand, chronic inflammation [44] and pain exacerbation [45] reflected by elevated C-reactive protein can act synergistically—the former through pro-inflammatory factor release exacerbating the postoperative inflammatory response [46], and the latter impeding functional recovery, which together prolong LOS [45].

The effect of these factors on LOS is consistent with previous studies, and the mechanism of the impact of these factors on LOS is explained by drawing on existing studies. However, no study has explicitly stated that prothrombin time and prothrombin activity affect LOS; on the contrary, this was confirmed in our study. Both thrombin time and prothrombin activity prolong LOS through complications, but they are fundamentally different. Precisely, the thrombin time mainly reflects the efficiency of converting fibrinogen to fibrin, and its prolongation indicates a decrease in fibrin production [47], in which case bleeding is a common complication. The complications represented by bleeding prolong LOS, but the impact of these complications on LOS is limited. Taking bleeding symptoms as an example, fibrin infusion can effectively reduce the amount of bleeding [48]. Decreased prothrombin activity is associated with systemic pathological conditions such as coagulation factor deficiency [49], hepatic insufficiency [47], jaundice [50], albuminemia [51], etc., and multidimensional treatment is usually required. This treatment is characterized by a long duration, which significantly prolongs LOS. Therefore, in our results, compared with the effect of thrombin time on LOS, prothrombin activity had a more significant impact on LOS.

The observed LOS (12.01 ± 4.28 days) exceeded contemporary benchmarks [23,24], likely reflecting institutional ERAS protocol limitations and cultural factors (such as the “Injured in the sinews or bones for one hundred days” concept) prolonging postoperative observation. Future work will optimize culturally adapted ERAS frameworks.

Study limitations included (1) a retrospective design with inherent selection bias [52]; (2) single-center data from Western China (2018–2024) limiting generalizability; and (3) that this study did not systematically assess the effect of preoperative albumin level on LOS. Although previous studies have suggested that the higher incidence of postoperative complications in patients due to lower albumin [29] may indirectly prolong LOS, the potential confounding effect of preoperative albumin needs to be further validated by subsequent prospective studies due to the scope of data collection in this study. Future prospective multicenter studies with expanded biochemical profiling are warranted.

## 5. Conclusions

The results of this retrospective study suggest that several factors (right lower extremity strength, ADL score, C-reactive protein, thrombin time, prothrombin activity, age, and ESR) directly or indirectly influence the LOS in patients undergoing primary THA according to the ERAS concept. These findings demonstrate clinical relevance for optimizing individualized care plans through preoperative risk stratification.

## Figures and Tables

**Table 1:** Detailed description of statistical values and univariate analysis.

Factors		Scale (%)	Statistic	*p*
Age	Less than 41 Years	65 (16.00)	1.958 ^a^	0.006 *
	41–60 Years	245 (60.00)		
	Older than 60 Years	98 (24.00)		
Right Lower Extremity Strength	Outside the Reference Range	109 (26.72)	2.234 ^b^	0.029 *
	Reference Range	296 (72.54)		
	Missing	3 (0.74)		
ADL score	100	90 (22.06)	3.538 ^a^	0.03 *
	61–99	271 (66.42)		
	41–60	25 (6.13)		
	Less than 41	14 (3.43)		
	Missing	8 (1.96)		
C-reactive Protein	Reference Range	330 (80.88)	−2.514 ^b^	0.016 *
	Outside the Reference Range	61 (14.95)		
	Missing	17 (4.17)		
Thrombin Time	Reference Range	124 (30.39)	−2.393 ^b^	0.019 *
	Outside the Reference Range	278 (68.14)		
	Missing	6 (1.47)		
Prothrombin Activity	Reference Range	364 (89.22)	2.582 ^b^	0.013 *
	Outside the Reference Range	38 (9.31)		
	Missing	6 (1.47)		
ESR	Reference Range	304 (74.51)	−2.519 ^b^	0.015 *
	Outside the Reference Range	94 (23.04)		
	Missing	10 (2.45)		

Note: ^a^ (F); ^b^ (t); * statistically significant.

**Table 2:** Multiple LRA of factors influencing LOS in primary THA patients (n = 408).

Item	Bias Regression Coefficient	Standard Error	Standard Regression Coefficient	*t*	*p*
Constant	32.232	20.832		1.547	0.123
Right Lower Extremity Strength	1.203	0.431	0.134	2.794	0.005
ADL score	−0.046	0.013	−0.182	−3.481	0.001
ESR	0.040	0.016	0.149	2.589	0.010

Note: *R*^2^ = 0.191, adjusted *R*^2^ = 0.131, F = 3.199, *p* < 0.05.

## Data Availability

Data presented in this study are available on request from the corresponding author due to privacy or ethical restrictions.

## Supplementary Materials

The following supporting information can be downloaded at https://www.mdpi.com/article/10.3390/healthcare13070777/s1. Table S1: Patient Characteristics.
