# Supplementary material for: Bayesian Analysis of Length of Stay Determinants in ERAS-Guided Hip Arthroplasty
Source: Healthcare (Basel). 2025 Mar 31;13(7):777. doi: 10.3390/healthcare13070777 (PMC11989033; doi:10.3390/healthcare13070777)
Supplement: Supplementary file 1 [file healthcare-13-00777-s001.zip › healthcare-3525535-supplementary.pdf]

Table S1: Patient Characteristics

Factors		Scale (%)	Univariate analysis		Multifactorial Analysis	
			**Statistic**	***P***	***t***	***P***
Age	Less 41 Years	65 (16.0)	1.958 [a]	0.006*	0.250	0.803
	Less 61 Years	245 (60.0)				
	Older than 60 Years	98 (24.0)				
Sex	Male	204 (50.0)	0.237 [b]	0.813	0.357	0.722
	Female	204 (50.0)				
Left Upper Extremity Strength	Reference Range	404 (99.0)	0.859	0.355	-1.671	0.096
	Outside the Reference Range	1 (0.2)				
	Missing	3 (0.7)				
Right Upper Extremity Strength	Reference Range	403 (98.8)	0.745 [b]	0.459	1.137	0.189
	Outside the Reference Range	2 (0.5)				
	Missing	3 (0.7)				
Left Lower Extremity Strength	Reference Range	309 (75.7)	-0.649 [b]	0.518	-0.875	0.382
	Outside the Reference Range	96 (23.5)				
	Missing	3 (0.7)				
Fall Hazard Score	Reference Range	283 (69.4)	-0.523 [b]	0.602	-0.963	0.336
	Outside the Reference Range	118 (28.9)				
	Missing	7 (1.7)				
Branden Score	Reference Range	248 (60.8)	-0.337 [b]	0.738	1.115	0.266
	Outside the Reference Range	9 (2.2)				
	Missing	151 (37.0)				
Gravity of urine	Reference Range	333 (81.6)	-0.695 [b]	0.490	-1.603	0.110
	Outside the Reference Range	42 (10.3)				
	Missing	33 (8.1)				
International Normalized Ratio (INR)	Reference Range	401 (98.3)	0.222 [a]	0.638	0.569	0.570
	Outside the Reference Range	1 (0.2)				
	Missing	6 (1.5)				

Interleukin 6	Reference Range	196 (48.1)	1.802 [a]	0.181	-1.168	0.244
	Outside the Reference Range	63 (15.4)				
	Missing	149 (36.5)				
Glucose	Reference Range	322 (78.9)	0.258 [b]	0.797	0.921	0.358
	Outside the Reference Range	83 (20.3)				
	Missing	3 (0.7)				
Creatinine	Reference Range	362 (88.7)	-1.200 [b]	0.234	1.095	0.274
	Outside the Reference Range	43 (10.5)				
	Missing	3 (0.7)				
D-dimer	Reference Range	121 (29.7)	0.731 [b]	0.468	-2.035	0.343
	Outside the Reference Range	120 (29.4)				
	Missing	167 (40.9)				
WBC	Reference Range	356 (87.3)	0.691 [b]	0.492	-0.193	0.847
	Outside the Reference Range	42 (10.3)				
	Missing	10 (2.5)				
RBC	Reference Range	350 (85.8)	-0.419 [b]	0.677	0.297	0.767
	Outside the Reference Range	48 (11.8)				
	Missing	10 (2.5)				
Haemoglobin	Reference Range	297 (72.8)	0.856 [b]	0.395	-0.463	0.644
	Outside the Reference Range	101 (24.8)				
	Missing	10 (2.5)				
Platelets	Reference Range	347 85.0)	-.0677 [b]	0.500	-0.153	0.878
	Outside the Reference Range	51 (12.5)				
	Missing	10 (2.5)				
Carbamide	Reference Range	371 (90.9)	-0.722 [b]	0.473	-0.018	0.986
	Outside the Reference Range	34 (8.3)				
	Missing	3 (0.7)				
Uric acid	Reference Range	313 (76.7)	0.289 [b]	0.773	1.170	0.243
	Outside the Reference Range	92 (22.5)				

	Missing	3 (0.7)				
Potassium	Reference Range	118 (28.9)	0.966 [b]	0.337	-1.137	0.189
	Outside the Reference Range	287 (70.3)				
	Missing	3 (0.7)				
Total Protein	Reference Range	373 (91.4)	0.153 [b]	0.879	-1.208	0.228
	Outside the Reference Range	32 (7.8)				
	Missing	3 (0.7)				
Right Lower Extremity Strength	Outside the Reference Range	109 (26.72)	2.234 [b]	0.029*	2.794	0.005*
	Reference Range	296 (72.54)				
	Missing	3 (0.74)				
ADL score	100	90 (22.06)	3.538 [a]	0.03*	-3.481	0.001*
	Less 100	271 (66.42)				
	Less 61	25 (6.13)				
	Less 41	14 (3.43)				
	Missing	8 (1.96)				
C-reactive Protein	Reference Range	330 (80.88)	-2.514 [b]	0.016*	-0.195	0.845
	Outside the Reference Range	61 (14.95)				
	Missing	17 (4.17)				
Prothrombin Activity	Reference Range	364 (89.22)	2.582 [b]	0.013*	-0.671	0.503
	Outside the Reference Range	38 (9.31)				
	Missing	6 (1.47)				
Thrombin Time	Reference Range	124 (30.39)	-2.393 [b]	0.019*	0.462	0.644
	Outside the Reference Range	278 (68.14)				
	Missing	6 (1.47)				
ESR	Reference Range	304 (74.51)	-2.519 [b]	0.015*	2.589	0.010*
	Outside the Reference Range	94 (23.04)				
	Missing	10 (2.45)				

Note: a (F); b (t); * (statistically significant)
